# Glycerine drives acute lung‐function impairment and airway inflammation after e‐cigarette aerosol exposure in mice

**DOI:** 10.1113/EP094175

**Published:** 2026-10-22

**Authors:** Alexander N. Larcombe, Maxine A. Janka, Luke J. Berry, Benjamin J. Mullins

**Affiliations:** ^1^ Occupation, Environment and Safety, School of Population Health Curtin University Bentley Western Australia Australia; ^2^ Wal‐yan Respiratory Research Centre The Kids Research Institute Australia Nedlands Western Australia Australia

**Keywords:** airway inflammation, asthma, electronic cigarette, forced oscillation technique, glycerin, propylene glycol, vaping

## Abstract

Electronic cigarettes generate an aerosol by heating an e‐liquid based on propylene glycol (PG) and/or glycerin (VG), with or without nicotine and flavourings. Their acute respiratory effects, particularly with respect to asthma, remain poorly defined. We exposed ovalbumin‐sensitised female BALB/c mice, which were subsequently challenged with ovalbumin (‘asthmatic’) or saline (‘non‐asthmatic’), to one of four e‐cigarette aerosols (PG or VG, each with 0 or 12 mg/mL nicotine) for 30 s. Airway resistance (*R*_aw_), tissue damping (*G*) and tissue elastance (*H*) were measured once/minute for 60 min using the forced oscillation technique. A second e‐cigarette aerosol was given at the 30‐min mark. Bronchoalveolar lavage (BAL) was taken for assessment of cellular inflammation. Group sizes were 4–9 mice (*n* = 53 for lung mechanics and 57 for BAL). All aerosols acutely increased *R*_aw_, *G* and *H*, and excipient was the dominant determinant. VG produced substantially larger increases than PG across most parameters and exposures (*P* < 0.0001, except airway resistance after the first exposure, *P* = 0.0569), responses approximately doubled after a second exposure, and allergic sensitisation augmented the repeated‐exposure mechanical response. Nicotine was not required for these effects and, where significant, attenuated them. In BAL, granulocytic inflammation was confined to asthmatic mice and was greater with VG than PG. Acute e‐cigarette aerosol exposure is not innocuous; e‐liquid composition, especially the excipient, is a key determinant of acute respiratory effects independent of nicotine.

## Abstract (highlights)

- **What is the central question of this study?**
  Does acute electronic cigarette aerosol exposure impair lung mechanics and/or provoke airway inflammation in a mouse model of asthma, and do these effects depend on the e‐liquid excipient (propylene glycol vs. glycerin) or on nicotine?
- **What is the main finding and its importance?**
  Brief e‐cigarette aerosol exposure acutely impaired lung mechanics and, in asthmatic mice, was associated with greater granulocytic airway inflammation. The excipient was the dominant determinant, with glycerin‐based aerosol producing substantially larger effects than propylene glycol‐based aerosol. These effects occurred largely independently of nicotine.

## 1 INTRODUCTION

Electronic cigarettes (‘e‐cigarettes’) heat and aerosolise an e‐liquid based on the humectants propylene glycol (PG) and/or glycerin (VG), with or without nicotine and flavourings, producing an aerosol that is inhaled (Lee et al., 2020). Their use has risen rapidly in many places, including among adolescents and people with asthma, and adolescents with asthma are more likely to vape than those without asthma (Han & Chung, 2021; Li et al., 2022). Despite this, the acute respiratory consequences of vaping remain incompletely defined (Hofmann et al., 2024).

Controlled human studies generally show that acute e‐cigarette use increases airway resistance, although spirometric indices are largely unchanged (Honeycutt et al., 2022). The picture is not uniform, however, as a controlled trial of nicotine‐ and flavour‐free aerosol found no significant change in lung function in either healthy or asthmatic volunteers after a 1‐h vaping session (Boulay et al., 2017). This implies that response may depend on dose or on specific aerosol constituents, with aerosolised humectants and their thermal‐degradation products being likely contributors (Sun et al., 2025). But even this is not clear‐cut. For example, inhaled PG mist has been shown to cause upper airway irritation, and in some subjects a slight reduction in FEV_1_/FVC plus a small increase in dyspnoea (Wieslander et al., 2001). However, a later human study found no exposure‐related changes in objective respiratory function under the tested acute conditions (Dalton et al., 2018). Glycerin (VG) generates a higher aerosol mass than PG (higher PM_2.5_) (Li et al., 2020), potentially delivering a larger dose of inhaled material to the airways. VG has also been implicated in case reports of vaping‐associated lung injury (McCauley et al., 2012), but its specific contribution to acute functional impairment remains uncertain. Nicotine, the main pharmacologically active constituent, can elicit bronchoconstriction (Hansson et al., 1994) but has also been reported to exert anti‐inflammatory actions (Blanchet et al., 2005), leaving its overall respiratory impact uncertain. Whether e‐cigarette aerosol exacerbates asthma, and which components are responsible, remains unresolved (Agache et al., 2024; Lee et al., 2024).

In this study we used a mouse model of allergic airways disease, and assessment of lung function using the forced‐oscillation technique, to investigate whether acute e‐cigarette aerosol exposure alters lung mechanics and airway inflammation, and whether any effect depends on the excipient (PG vs. VG), nicotine (0 vs. 12 mg/mL) and/or allergic sensitisation status. We hypothesised that acute exposure would elicit impairments in lung function that would be greater in sensitised animals and greater for VG‐based e‐liquids and those containing nicotine.

## 2 METHODS

### 2.1 Ethical approval

All procedures were approved by The Kids Research Institute Australia Animal Ethics Committee (approval #271) and complied with the *Australian Code for the Care and Use of Animals for Scientific Purposes* (8th Edition, 2013). The authors understand the ethical principles under which *Experimental Physiology* operates, and this work complies with the journal's animal ethics checklist.

### 2.2 Mice and treatment groups

Eight‐week‐old female BALB/c mice were obtained from the Animal Resource Centre (Murdoch, Western Australia) and housed in individually ventilated cages (Sealsafe, Tecniplast, Buguggiate, Italy) on non‐allergic, dust‐free bedding. All mice had ad libitum access to water and an allergen‐free diet (Specialty Feeds, Glen Forrest, WA, Australia). The experimental unit (*n*) was the individual mouse. Throughout the main text, tables and figure legends, *n* is the number of individual mice, with each mouse contributing a single value to each analysis, so that *n* is not inflated by repeated measurements within an animal. Two mice were excluded from all analyses on the basis of the record made at the time of the experiment: one was in poor condition and one died on the ventilator before the protocol was complete. Mice were allocated across eight treatment groups defined by sensitisation status, excipient and nicotine concentration (Table 1). Two further non‐asthmatic mice in the 0PG group were lavaged but have no lung‐function recording, which is why *n* differs between the two outcomes in that group. Final group sizes ranged from *n* = 4 to 9 for the lung‐function analyses and *n* = 5 to 9 for bronchoalveolar lavage. In total, *n* = 53 mice contributed lung‐function data and *n* = 57 mice contributed bronchoalveolar lavage data; per‐group *n* for both outcomes are given in Table 1.

**TABLE 1:** Study groups, sample sizes and baseline body weight.

Group	Sensitisation	Excipient	Nicotine (mg/mL)	*n* (lung function)	*n* (BAL)	Body weight ± SD (g)
0PG	Non‐asthmatic	PG	0	6	9	18.0 ± 1.2
12PG	Non‐asthmatic	PG	12	8	8	17.5 ± 0.7
0VG	Non‐asthmatic	VG	0	8	8	17.4 ± 0.9
12VG	Non‐asthmatic	VG	12	9	9	18.4 ± 0.7
0PG	Asthmatic	PG	0	6	6	18.9 ± 0.8
12PG	Asthmatic	PG	12	6	6	19.3 ± 0.9
0VG	Asthmatic	VG	0	6	6	19.5 ± 1.3
12VG	Asthmatic	VG	12	4	5	19.8 ± 1.2

Each group was defined by allergen challenge (asthmatic: OVA‐challenged; non‐asthmatic: saline‐challenged), e‐liquid excipient (PG, propylene glycol; VG, glycerin) and nicotine concentration. Body weight is mean ± SD; *n* differs between lung‐function and BAL analyses because different subsets yielded analysable data.

### 2.3 Sensitisation and challenge protocol

Mice were sensitised to ovalbumin (OVA) using a standard OVA/alum protocol (Zosky et al., 2008). Briefly, mice were sensitised on day 0 and boosted on day 14 with intraperitoneal injections of 20 µg of chicken egg OVA (Sigma, St Louis, MO, USA) suspended in 200 µL of Alum (Alu‐gel‐S, Serva, Heidelberg, Germany). Mice were then challenged with a 30‐min aerosol of 1% OVA (‘asthmatic’) or saline (‘non‐asthmatic’) on day 21.

### 2.4 E‐cigarette aerosol exposure and lung function

Twenty‐four hours after challenge, mice were anaesthetised by intraperitoneal injection of a ketamine/xylazine mixture (final concentrations 40 mg/mL ketamine and 2 mg/mL xylazine; Troy Laboratories, New South Wales, Australia) at 0.1 mL per 10 g body weight, giving 400 mg/kg ketamine and 20 mg/kg xylazine; approximately one‐third of the dose was given to induce surgical anaesthesia for the tracheostomy and the remaining two‐thirds once the animal was connected to the ventilator, with top‐ups of one‐third of the original dose as required to maintain general anaesthesia throughout the ∼60‐min non‐recovery protocol. Depth of anaesthesia was confirmed by loss of the pedal withdrawal reflex and loss of response to stimuli and monitored thereafter by the absence of spontaneous respiratory effort and of any response to tactile stimulation. No neuromuscular blocking agents were used. Mice were tracheostomised and a 10 mm‐long polyethylene cannula inserted into the trachea. This was secured using surgical silk prior to mice being mechanically ventilated (flexiVent, SCIREQ, Montreal, Canada) as previously described (Zosky et al., 2008). After lung volume history was standardised, lung function at functional residual capacity was measured using the forced oscillation technique (FOT). Mice then received a 10‐s saline aerosol, and mechanics were measured again before one of four e‐cigarette aerosols was delivered. Aerosol was generated with an Innokin iTaste MVP device, drawn into a 50 mL syringe and pumped slowly into the inspiratory line over ∼10 s; this was repeated three times at ∼10‐s intervals (∼30 s of aerosol delivery in total, over ∼1 min). Respiratory mechanics were then measured by FOT once per minute for 30 min, after which a second exposure was delivered and FOT continued for a further 30 min. Airway resistance (Newtonian resistance, which is equivalent to airway resistance in mice; *R*_aw_), tissue damping (*G*) and tissue elastance (*H*) were derived from the constant‐phase model (Hantos et al., 1992).

The four e‐liquids (Mt Baker Vapor, Lynden, WA, USA) were the same as those used previously (Larcombe et al., 2017). All were ‘American Tobacco’ flavour and differed only in nicotine content (0 or 12 mg/mL) and main excipient, giving four varieties: 0PG, 12PG, 0VG and 12VG. Gas chromatography–mass spectrometry of the e‐liquids, reported previously (Larcombe et al., 2017), confirmed that the PG‐ and VG‐based e‐liquids were chemically distinct: both PG‐based aerosols were 100% propylene glycol, whereas the VG‐based e‐liquids were predominantly glycerin with small amounts of propylene glycol (0VG, 95.30% glycerin and 4.70% propylene glycol; 12VG, 97.53% glycerin and 2.47% propylene glycol).

### 2.5 Bronchoalveolar lavage

Following measurements, the lungs were lavaged by washing 0.5 mL of saline in and out of the lungs three times through the tracheal cannula. At the end of the protocol, while still under general anaesthesia, mice were euthanised by cervical dislocation. Total cell counts were obtained with a haemocytometer and differential counts performed on 300 cells where possible, stained with Rapid Stain (Amber Scientific, Midvale, Western Australia) by light microscopy; absolute numbers of each cell type per mL were calculated as the total count multiplied by its proportion.

### 2.6 Statistical analysis

Traces were aligned to the protocol clock (baseline, saline, then 30‐min exposure windows) so that the onset of the second exposure coincided across animals. For each mouse, the response to each exposure was summarised as the mean *R*_aw_, *G* or *H* over that 30‐min window, expressed as the percentage change from that animal's own saline value. Summary values were analysed by three‐way analysis of variance (ANOVA) using Type II sums of squares, with sensitisation status, excipient and nicotine as factors, performed separately for each parameter and exposure window. Bronchoalveolar lavage cell counts were log_10_(*x* + 1) transformed before analysis to satisfy the assumptions of normality and equal variance; the lung‐function summary values met these assumptions untransformed and were analysed on their original scale. Where a sensitisation × excipient interaction was present, the excipient effect was examined within each sensitisation stratum by two‐way ANOVA. Eosinophils were undetectable in all non‐asthmatic mice, so for that cell type we base our interpretation on the within‐asthmatic analysis. One non‐asthmatic mouse had no usable post‐saline measurement and was referenced to its own pre‐saline baseline; *n* was 53 mice for lung function and 57 for lavage. In every analysis the unit of replication was the mouse, with one value per mouse per outcome, so that no animal contributed more than one observation to any test. As a sensitivity analysis, the per‐minute data were also fitted with linear mixed‐effects models (sensitisation, excipient, nicotine and minute as fixed effects), first with a random intercept per animal and then with an additional random slope for minute to allow for within‐animal temporal drift. Baseline body weights were compared by ANOVA. Analyses were performed in Python (statsmodels); *P* < 0.05 was considered significant. Data are presented as means ± SD. Exact *P*‐values for all effects are given in Table 2. For display, figure traces were smoothed with a 5‐point moving average applied within each exposure window. The complete per‐animal dataset is provided as Supporting Information Data S1 and S2.

**TABLE 2:** Exact *P*‐values from three‐way ANOVA (Type II sums of squares), to three significant figures.

Outcome	Sens	Exc	Nic	Sens × Exc	Sens × Nic	Exc × Nic	Sens × Exc × Nic
*R*_aw_, exposure 1	0.411	0.0569	0.00279	0.169	0.873	0.495	0.543
*R*_aw_, exposure 2	0.00130	<0.0001	0.00652	0.200	0.301	0.887	0.159
*G*, exposure 1	0.226	<0.0001	0.0217	0.248	0.356	0.235	0.399
*G*, exposure 2	0.0650	<0.0001	0.000219	0.422	0.550	0.110	0.192
*H*, exposure 1	0.0572	<0.0001	0.0604	0.226	0.226	0.260	0.778
*H*, exposure 2	0.00578	<0.0001	0.000305	0.455	0.571	0.0968	0.0593
Macrophages	0.0438	0.00396	0.738	0.993	0.552	0.338	0.978
Neutrophils	<0.0001	0.512	0.427	0.00173	0.653	0.911	0.604
Eosinophils	<0.0001	0.0249	0.556	0.00727	0.482	0.604	0.528

Lung‐function outcomes are the percentage change from each animal's own saline value averaged over the exposure window (*n* = 53 mice); lavage counts were log_10_(*x* + 1) transformed (*n* = 57 mice). Sens, sensitisation; Exc, excipient; Nic, nicotine. Per‐group *n* are given in Table 1.

## 3 RESULTS

### 3.1 Weight

Body weight did not differ among the four e‐cigarette groups within either sensitisation status (non‐asthmatic, *P* = 0.0917; asthmatic, *P* = 0.5450; Table 1). Asthmatic mice were marginally heavier than non‐asthmatic mice (19.3 ± 1.0 vs. 17.8 ± 0.9 g; *P* < 0.0001). Body weight was recorded for every mouse in the study (*n* = 57 mice; 34 non‐asthmatic and 23 asthmatic), with per‐group *n* as given in Table 1.

### 3.2 Respiratory mechanics

All four e‐cigarette aerosols acutely increased airway resistance (*R*_aw_), tissue damping (*G*) and tissue elastance (*H*) above saline values (Figure 1). Lung mechanics were analysed in *n* = 53 mice (*n* = 4–9 mice per group; Table 1), each animal contributing one summary value per exposure window. The excipient was the dominant determinant of the response. VG‐based aerosols produced larger increases than PG‐based aerosols for all three parameters and at both exposures, and this difference was statistically significant in every case except *R*_aw_ after the first exposure. After the first exposure, VG raised *R*_aw_, *G* and *H* by 23.7%, 31.9% and 36.8% above saline, respectively, compared with ∼15%, 14% and 17% for PG. The excipient effect was significant for the tissue parameters (*G* and *H*, both *P* < 0.0001) but did not reach significance for *R*_aw_ (*P* = 0.0569). The divergence widened and was significant for all three parameters after the second exposure, when VG increased *R*_aw_, *G* and *H* even further (by 51.0%, 78.7% and 106.4%, vs. 23.7%, 31.8% and 43.5% for PG). The response was also cumulative with increases following the second exposure being roughly double those following the first. Allergic sensitisation modified this response, but principally at the second exposure, where sensitised and challenged (‘asthmatic’) mice showed greater increases than non‐asthmatic mice for *R*_aw_ (*P* = 0.00130) and *H* (*P* = 0.00578), but not *G* (*P* = 0.0650); first‐exposure differences were not significant (all *P* ≥ 0.0572; Table 2). In contrast, nicotine was not required for any of these effects. Where it reached significance, nicotine was associated with an attenuated response (12 mg/mL lower than 0 mg/mL; e.g., *R*_aw_, *P* = 0.00279 and *P* = 0.00652 after the first and second exposures; *G*, *P* = 0.0217 and *P* = 0.000219; *H*, *P* = 0.0604 and *P* = 0.000305). No interaction reached significance (all *P* ≥ 0.0593; Table 2), indicating that the mechanical impairment was driven by the aerosol vehicle rather than by nicotine.

**FIGURE 1:**
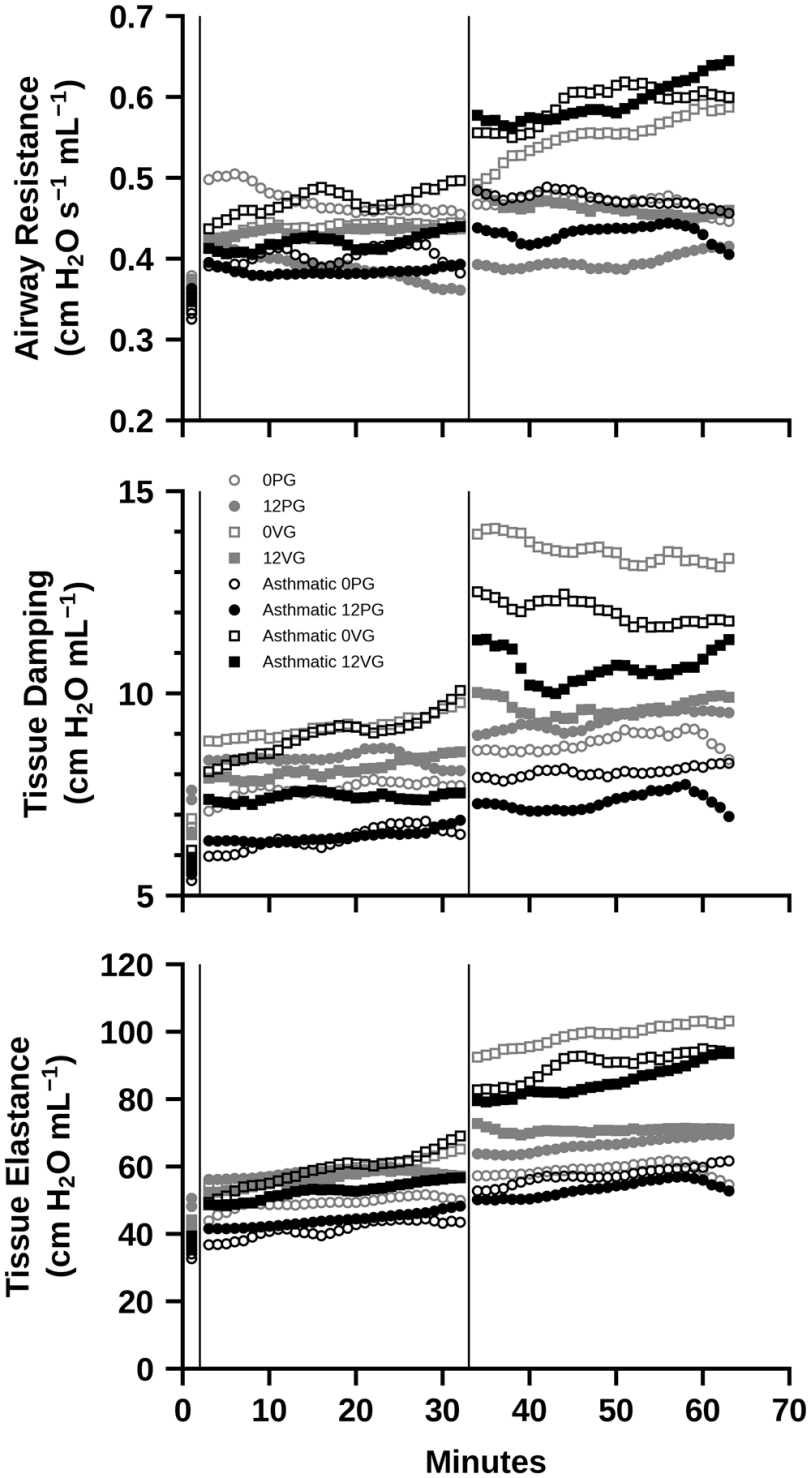
Respiratory mechanics during acute e‐cigarette aerosol exposure in asthmatic and non‐asthmatic mice. Airway resistance (*R*_aw_, top), tissue damping (*G*, middle) and tissue elastance (*H*, bottom) were measured by the forced oscillation technique once per minute. After a baseline reading and a saline aerosol (minute 0), mice received a first e‐cigarette aerosol exposure and a second exposure 30 min later (represented by vertical black lines). Aerosols were generated from propylene glycol (PG; circles) or glycerin (VG; squares) e‐liquid containing 0 (open shapes) or 12 mg/mL nicotine (closed shapes). Lines are group means smoothed with a 5‐point moving average within each exposure window; *n* is the number of mice (non‐asthmatic: 0PG 6, 12PG 8, 0VG 8, 12VG 9; asthmatic: 0PG 6, 12PG 6, 0VG 6, 12VG 4); asthmatic groups are shown in black and non‐asthmatic groups in grey. Each mouse contributed one measurement per time point, and statistical comparisons used a single summary value per mouse per exposure window (total *n* = 53 mice).

### 3.3 Airway inflammation

Bronchoalveolar lavage was analysed in *n* = 57 mice (*n* = 5–9 mice per group; Table 1), with a single lavage sample per mouse. Macrophages predominated in BAL and were more numerous with VG (*P* = 0.00396) and with allergic sensitisation (*P* = 0.0438; Figure 2), with no effect of nicotine (*P* = 0.738) and no significant interaction (all *P* ≥ 0.338; Table 2). Neutrophils and eosinophils were essentially confined to asthmatic mice (*P* < 0.0001 for both), with significant sensitisation‐by‐excipient interactions (neutrophils, *P* = 0.00173; eosinophils, *P* = 0.00727). Within asthmatic mice, VG‐based aerosols increased both neutrophils (*P* = 0.0406) and eosinophils (*P* = 0.0351) with no effect of nicotine (*P* = 0.389 and *P* = 0.568) and no interaction (*P* = 0.632 and *P* = 0.614); the asthmatic 0PG group had the lowest granulocyte counts. Eosinophils were undetectable in all non‐asthmatic mice.

**FIGURE 2:**
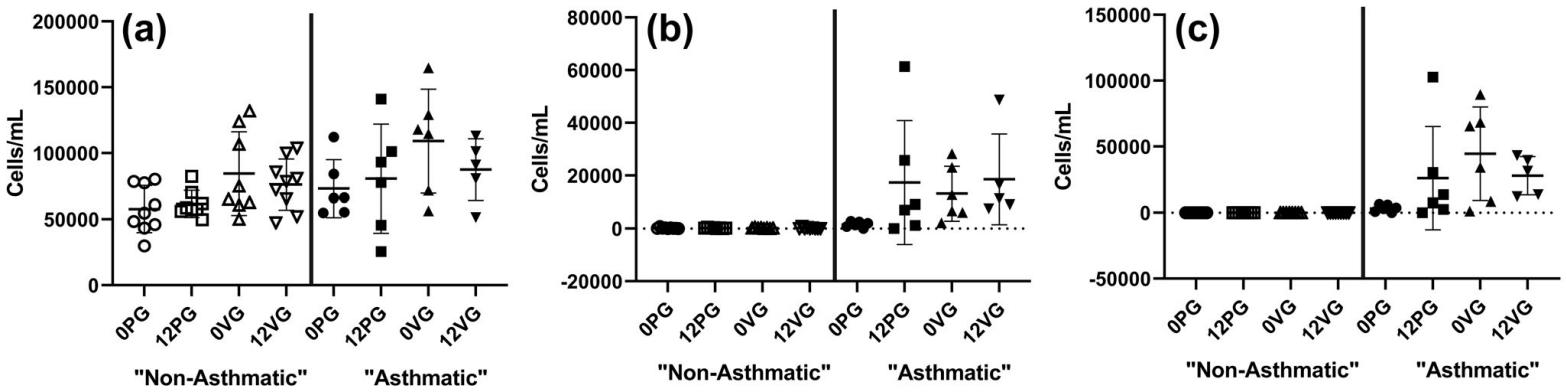
Bronchoalveolar lavage inflammation after acute e‐cigarette aerosol exposure. Absolute numbers of macrophages (a), neutrophils (b) and eosinophils (c) in asthmatic and non‐asthmatic mice exposed to propylene glycol (PG) or glycerin (VG) e‐liquid containing 0 or 12 mg/mL nicotine. Note different scales. Data are means ± SD with individual mice shown as symbols. *n* is the number of individual mice, each contributing a single lavage sample (total *n* = 57 mice; non‐asthmatic: 0PG 9, 12PG 8, 0VG 8, 12VG 9; asthmatic: 0PG 6, 12PG 6, 0VG 6, 12VG 5). Exact *P*‐values are given in Table 2.

## 4 DISCUSSION

The key finding of this study was that acute exposure to e‐cigarette aerosol was not benign but instead resulted in transient impairment of lung mechanics in all groups. In OVA‐sensitised and challenged ‘asthmatic’ mice, the glycerin excipient was also associated with greater granulocytic airway inflammation than propylene glycol. Importantly, it was the excipient, rather than the presence or absence of nicotine, that was the dominant determinant of effect, with VG consistently producing larger responses than PG.

This excipient effect is consistent with the physicochemical behaviour of VG aerosols, which carry a substantially greater particulate mass than PG (Li et al., 2020) and therefore can potentially deliver a larger dose to the airways. Beyond dose, glycerin engages the airway lipid system in a way that PG does not. For example, VG‐containing aerosols, but not PG alone, reduce epithelial cystic fibrosis transmembrane conductance regulator (CFTR) activity by partitioning into and stiffening the cell membrane and concurrently increase mucus concentration and inflammatory mediator expression, while the VG also disrupts surfactant phospholipid homeostasis and the function of resident alveolar macrophages (Kim et al., 2022; Madison et al., 2019). Both effects occur independently of nicotine, consistent with the limited role of nicotine in our own data. The comparatively modest and more transient effects of PG aerosols may reflect their lower aerosol mass and more rapid clearance (Li et al., 2020), together with the absence of this glycerin‐specific interaction with the airway lipid and surfactant system. The near‐doubling of responses after a second brief exposure indicates that even short, repeated use can have a cumulative impact on lung mechanics, in keeping with the dose‐dependence of vaping‐related changes in airway resistance reported in humans (Honeycutt et al., 2022).

An important consideration is whether the measured changes in mechanics reflect deposited aerosol within the airways rather than an alteration in the airway tissue itself. Both humectants deposit as a hygroscopic liquid, and a liquid film lining the lumen can raise airway resistance by narrowing the airway and raise *G* and *H* by altering surface tension, promoting airway closure and derecruiting lung units. A contribution from deposited aerosol is therefore likely. Several features of the data nonetheless argue against a purely transient physical effect: mechanics did not recover over the 30 min after either exposure, the second response was approximately double the first, and the magnitude depended on sensitisation status and nicotine content, neither of which alters the physical characteristics of the aerosol. The distinction is in any case not absolute, since for VG the interaction between deposited glycerin and the airway lining is the proposed mechanism rather than a confounder of it. Resolving the two would require measurement of the deposited dose and a longer recovery period than this protocol allowed.

Most prior in vivo models that have assessed lung function have used fixed PG/VG blends delivered over days to weeks (Chapman et al., 2019; Szafran et al., 2020). Such repeated‐exposure protocols show that PG/VG aerosols can drive airway inflammation, mucus dysregulation and altered responsiveness even in the absence of nicotine. The high cumulative doses, prolonged durations and fixed excipient ratios they employ make it difficult to attribute effects to any single e‐liquid component, and they do not capture the immediate, early‐phase mechanical response to a single exposure that is the focus here. We previously found that glycerin‐based aerosols caused mice to be hyper‐responsive to methacholine independently of nicotine (Larcombe et al., 2017). This supports the present data, which show that this excipient hierarchy is already evident in the acute early‐phase response. That clear excipient effects emerged here, whereas a controlled human trial of vehicle‐only aerosol detected none (Boulay et al., 2017), likely reflects the higher delivered dose and direct lower‐airway deposition achievable in an anaesthetised, ventilated preparation.

Nicotine at 12 mg/mL did not exacerbate lung‐function deficits and, where the effect was significant, attenuated them. This contrasts with the bronchoconstrictor potential of inhaled nicotine (Hansson et al., 1994) but is compatible with its recognised anti‐inflammatory actions (Blanchet et al., 2005), and with a murine allergic‐airways model in which nicotine‐containing e‐cigarettes suppressed airway inflammation (Chapman et al., 2019). A further possibility is that nicotine‐containing e‐liquids generate lower concentrations of the reactive carbonyl degradation products thought to drive aerosol toxicity (Sun et al., 2025). This finding warrants confirmation across a range of nicotine concentrations.

In our study, brief exposure altered lung mechanics, which agrees with controlled human data in which short e‐cigarette sessions acutely raise respiratory system resistance and impedance in both healthy and asthmatic individuals (Lappas et al., 2018). A comparable acute response has also been demonstrated in anaesthetised guinea pigs whereby a single puff of e‐cigarette aerosol evoked immediate, transient bronchoconstriction (Khosravi et al., 2018). In that study, the response was abolished by nicotine‐free aerosol and by cholinergic blockade, indicating a vagally mediated reflex triggered by nicotine. Our anaesthetised, tracheostomised and mechanically ventilated preparation largely bypasses this reflex arc, so a nicotine‐driven reflex would be expected to contribute little. That the excipient was instead the dominant determinant of the acute early‐phase response argues against a reflex mechanism and points to a direct interaction between the glycerin‐based aerosol and the airway tissue and surfactant system. To our knowledge, this is the first demonstration that excipient type governs the acute early‐phase response to e‐cigarette aerosol.

Allergic sensitisation amplified the mechanical response, particularly to repeated exposure, and was a prerequisite for neutrophil and eosinophil recruitment into the airways. Because this granulocytic inflammation was confined to sensitised, allergen‐challenged animals (*P* < 0.0001 for both cell types) and lavage was performed only ∼1 h after the first aerosol, too brief an interval for substantial *de novo* granulocyte recruitment into the airway lumen, the neutrophils and eosinophils recovered almost certainly reflect the preceding allergic challenge rather than direct aerosol‐induced recruitment (Tomkinson et al., 2001). The more modest excipient‐associated difference within asthmatic mice (greater with VG than PG) may instead reflect enhanced mobilisation or recovery of cells already resident in the airway wall (Tomkinson et al., 2001). Such an effect would agree with previous research showing that e‐cigarette aerosol can aggravate allergen‐induced airway inflammation (Taha et al., 2020) and can alter allergic airways disease, including airway hyper‐responsiveness, in an excipient, flavour and nicotine‐dependent manner (Chapman et al., 2019). Together with epidemiological evidence that people with asthma are both more likely to vape and more likely to experience asthma exacerbations (Agache et al., 2024), this supports the possibility that they are more susceptible to the acute respiratory effects of e‐cigarette aerosol than people without asthma.

Several limitations to our study should be noted. Exposures were acute and the study cannot address chronic effects, some sample sizes were small, a single nicotine concentration was tested, bronchoalveolar lavage was performed too soon after exposure to capture any cellular recruitment elicited by the aerosol itself, the design cannot separate deposited aerosol from an effect on the airway tissue, and extrapolation from mice to humans requires caution. Additionally, we did not perform bronchoprovocation testing. The protocol was designed to capture the early‐phase response and required uninterrupted measurement of mechanics for 60 min with each animal as its own saline control, which a methacholine dose–response challenge would have confounded. We accept that airway hyper‐responsiveness may be a more sensitive index of exposure than baseline mechanics. We summarised each animal's time course to a single value per window to avoid pseudoreplication; a linear mixed‐effects analysis of the per‐minute data reproduced every effect in direction and magnitude and agreed with the primary analysis in 15 of 18 main‐effect tests, the three exceptions all lying within 0.01 of the significance threshold and returning to non‐significance when within‐animal temporal drift was modelled. Thus, we are confident that, in our model, acute e‐cigarette aerosol exposure negatively impacts lung function, and e‐liquid composition (particularly the choice of excipient) is a key determinant of effect, independent of nicotine. E‐liquid composition should be considered in the risk assessment of e‐cigarettes and when advising people with asthma.

## AUTHOR CONTRIBUTIONS

Alexander N. Larcombe: Conceptualization, methodology, data curation, formal analysis, investigation, resources, validation, visualization, writing – original draft, writing – review & editing, funding acquisition, project administration, supervision. Maxine A. Janka: Conceptualization, methodology, investigation, writing – review & editing. Luke J. Berry: Conceptualization, methodology, investigation, writing – review & editing, funding acquisition. Benjamin J. Mullins: Conceptualization, resources, writing – review & editing, funding acquisition, project administration, supervision. All authors have read and approved the final version of this manuscript and agree to be accountable for all aspects of the work in ensuring that questions related to the accuracy or integrity of any part of the work are appropriately investigated and resolved. All persons designated as authors qualify for authorship, and all those who qualify for authorship are listed.

## CONFLICT OF INTEREST

The authors declare no conflicts of interest and have received no funding from any organisation with an interest in the manufacture or marketing of tobacco or e‐cigarette products.

## GENERATIVE AI STATEMENT

In preparing this manuscript the authors used a generative artificial‐intelligence assistant (Claude, Anthropic; model Opus 5.0, accessed June 2026) for the following tasks: (i) language editing and proofreading; (ii) calculation of descriptive summary statistics (body‐weight means and standard deviations, and per‐window percentage changes in lung function) from the authors' own raw data; and (iii) independent reproduction of the analysis of variance to verify the reported results. All experimental data were generated by the authors. Claude was not used to generate, fabricate or alter any primary data. All AI‐assisted text and calculations were verified against the source data and edited by the authors, who reviewed and approved the final wording and take full responsibility for the integrity, accuracy and content of the manuscript.

## Supporting information



Data S1. BAL cell counts.

Data S2. Lung function readings.

## Data Availability

All data underlying the figures and tables of this article are provided as Supporting Information.
